# Novosorb^®^ BTM- history, production and application in challenging wounds

**DOI:** 10.3389/fbioe.2024.1450973

**Published:** 2024-11-13

**Authors:** Rohan Rajaram, Min Zhang, Gehan Premaratne, Sally Ng

**Affiliations:** ^1^ Department of Plastic and Reconstructive Surgery, Austin Health, Heidelberg, VIC, Australia; ^2^ Department of Medicine, Dentistry and Health Sciences, The University of Melbourne, Melbourne, VIC, Australia; ^3^ Department of Surgery (Austin Precinct), The University of Melbourne, VIC, Australia

**Keywords:** biodegradable temporising matrix (BTM), burns, plastic and reconstructive surgery (PRS), necrotising fasciitis, chronic ulcer

## Abstract

Novosorb^®^ Biodegradable Temporising Matrix (BTM) is an entirely synthetic dermal matrix that is gaining popularity in the management of challenging wounds. Not only does it provide a framework in which to grow an organised neodermis, it is also especially resistant to infection. Today, the matrix is available as a 2 mm thick open cell polyurethane foam with a non-degrading sealing membrane. Its current form is the result of numerous *in vitro* and *in vivo* experiments that examined its shape, biodegradation, inflammatory response, and cytotoxicity. Clinical data on the use of BTM in a variety of cases is novel and presents early insights into its ability to foster wound healing where otherwise improbable. This review presents the history and development of Novosorb^®^ BTM as well as all the currently available clinical data on its efficacy in difficult wounds such as: major burns, necrotising soft tissue infection, chronic wounds and in non graftable wound beds.

## Introduction

Traditionally, management of large or complex soft tissue defects has relied on techniques such as autografts, local flap coverage or tissue transfer. However, associated limitations such as limited donor site availability and morbidity has underscored the need for alternative strategies.

Biological matrices, while initially promising, are susceptible to immunogenic reactions and disease transmission risks or cultural and ethical concerns regarding animal-derived products (Greenwood, 2016). Furthermore, their availability may be limited, and their integration with host tissues can be unpredictable (Greenwood, 2016). These shortcomings led to the development of synthetic dermal templates such as NovoSorb^®^ Biodegradable Temporising Matrix (BTM) (PolyNovo Biomaterials Pty Ltd., Port Melbourne, VIC, Australia).

The development of BTM began in 2004 at the Royal Adelaide Hospital in South Australia. This fully synthetic dermal matrix features a polyurethane bilayer consisting of a 2-mm thick biodegradable open cell foam and a non-biodegradable sealing membrane (Granick et al., 2023). The porous matrix facilitates cellular infiltration and serves as a scaffold for neodermis formation. The sealing membrane ensures physiological wound closure and contains microfenestrations to prevent debris accumulation (Granick et al., 2023).

Central to the functionality of BTM is its ability to serve as a temporising scaffold for cellular infiltration and proliferation while gradually degrading over time (PolyNovo, 2024), providing immediate wound coverage and structural support during the initial phases of wound healing. This feature is particularly relevant in cases where extensive tissue loss or complex wound geometries preclude primary closure or traditional reconstructive techniques. By facilitating wound stabilization and promoting granulation tissue formation, BTM creates an optimal environment for subsequent epithelialization and tissue remodelling. Its controlled degradation kinetics also allow for the gradual transfer of mechanical loads to the healing tissue and the stimulation of angiogenesis and collagen deposition (PolyNovo, 2024).

The versatility of BTM extends to its applicability in challenging clinical scenarios. In wounds prone to infection, BTM, as distinct from biological dermal matrices, does not provide biological components to encourage microbial growth and therefore may confer superior resistance to infection (Greenwood et al., 2018). Similarly, BTM provides a protective covering in defects involving exposed bone or tendon, fostering a conducive environment for tissue ingrowth, and preventing adhesion formation.

This review aims to consolidate existing knowledge and provide a comprehensive assessment of the development, efficacy, and clinical applications associated with NovoSorb^®^ BTM to elucidate its role as a valuable adjunct in armamentarium of reconstructive surgeons for complex wound management.

## History and production of Novosorb^®^ BTM

The Novosorb^®^ BTM commercially available today is the product of many fundamental conceptualisation, testing and iterative analyses which ensured its safety and efficacy. Initially, the ideal polymer base was investigated in both *in vitro* and *in vivo* analyses to determine tensile strength, time to degradation, toxicity of degradation end products and ability to foster keratinocyte infiltration and wound healing (Li et al., 2009). Polyurethanes are traditionally designed to be non-degradable. They are comprised of three main subunits: a polyol, a diisocyanate hard layer and a chain extender (Kollmannsberger et al., 2018). BTM polyurethanes alter this structure to use a biodegradable polyol known as polycaprolactone and use a degradable chain extender placed at strategic points within the polymer to control the rate of degradation (Lim et al., 2013). As such a host of degradable polyurethanes, which degrade *in vivo* via hydrolysis, were made available for testing.

Initial tests centred around the *in vitro* degradation rate and cytotoxicity of different structural arrangements of biodegradable polyurethanes. These polyurethanes differed in the arrangement and order of their subunits of polyol, diisocyanate and degradable chain extenders and were given the placeholder titles of BTM-1, BTM-2, and BTM-3 (Li et al., 2009). After undergoing the above *in vitro* tests, it was discovered that the BTM-2 arrangement was able to maintain structural integrity for long enough to encourage wound healing while also readily degrading from the physiological medium within 6 months. Furthermore, this arrangement of polyurethane was also found to exert the lowest cytotoxic burden onto an *in vitro* medium of keratinocytes, dermal fibroblasts, and microvascular endothelial cells. Encouragingly, there was no significant difference in cytotoxicity of the BTM-2 arrangement even when compared to existing synthetic degradable surgical tools such as Monocryl (Li et al., 2009).

Subsequent testing endeavoured to assess the susceptibility of BTM-2 to elicit a systemic inflammatory reaction as well as its ability to encourage the formation of a neodermis within rat and sheep *in vivo* models (Greenwood et al., 2010). Within rat models, BTM-2 was applied to a surgical wound and serum inflammatory markers were tracked at aggregated time points across 24 weeks. Furthermore, the local wound environment was also analysed with H&E staining and light microscopy (Greenwood et al., 2010). This analysis found that BTM-2 did not elicit a systemic inflammatory response as tracked by serum biomarkers. Furthermore, the local inflammatory reaction seen in BTM application was comparable to controls such as Prolene and Monocryl (Greenwood et al., 2010). As such, the ability for BTM-2 to facilitate wound healing was investigated in a sheep wound model over 29 days. When compared to a negative control and an Integra^®^ treated comparator, BTM-2 demonstrated a robust resistance to wound contracture, thereby allowing for the regeneration of a neodermis (Greenwood et al., 2010).

The next step in development of Novosorb^®^ BTM was the form in which BTM-2 would be integrated. Initial testing examined BTM-2 within a ‘spun mat’ form where six layers of cross hatched BTM-2 polymer were placed atop one another to create a three-dimensional lattice (Wagstaff et al., 2014). While nascent results from the ‘spun mat’ configuration were promising, an open cell foam form of BTM-2 eventually superseded the ‘spun mat’ form. This was due to the ability to better control cell and pore size while also utilising less polyurethane material, thereby reducing production costs (Wagstaff et al., 2014). The open foam configuration of BTM-2 was initially validated as a foam for negative pressure wound therapy where it demonstrated equitable safety in short term integration situations. Furthermore, the foam configuration of BTM-2 was also shown to facilitate the take of subsequent split skin grafting, albeit with issues of wound contracture and insensible fluid losses (Greenwood and Dearman, 2012). This prompted the optimisation of the foam configuration and the addition of a non-degradable polymer epidermal layer to contain granulation and neodermis formation, thereby reducing contraction.

The optimised foam structure that ultimately underwent human testing contained open cells connected by pores approximately 150 microns in size. This dermal layer was roofed by a non-degradable polyurethane epidermal layer. The aim of this structure is to compartmentalise a large wound into many smaller “micro-wounds” which change the biology by which the wound is healed. Within micro-wounds, it is purported that there is less cellular signalling to initiate scarring and contracture allegedly resulting in ‘Regenerative Wound Healing’ as compared to ‘Reparative Wound Healing’ seen in conventional wounds (PolyNovo, 2024).

## Wound healing with Novosorb^®^ BTM- true regeneration?

Novosorb^®^ BTM, like other dermal matrices, facilitates wound healing by providing a template through which angiogenesis and fibroblast migration can occur. This organises the efficient regrowth of a neodermis upon which a split thickness skin graft can be applied in four to 6 weeks’ time. By 6 months, experimental data demonstrates that almost all integrated BTM is expected to degrade via hydrolysis (Tatai, 2013). Clinically, this has been demonstrated in biopsies at 12–18 months (Wagstaff et al., 2015).

Wound healing generally occurs via a process known as reparative wound healing characterised by four biological stages. These are: haemostasis, inflammation, proliferation, and remodelling (Guo and Dipietro, 2010). In response to significant loss of dermis, presence of infection or fluid loss, wound healing can be hampered by severe contracture, scarring and loss of dermal architecture in an attempt to rapidly close the substantial wound (Guo and Dipietro, 2010).

By distinction, regenerative wound healing is a biological form of wound healing seen in foetal wounds until 24 weeks of gestation (Reinke and Sorg, 2012). It serves as the ideal goal for technologies aiming to improve wound healing. This is because regenerative wound healing results in a healed wound with virtually no scarring or contracture and renewed dermal appendages (Singer, 2022). The main biochemical differences seen between regenerative wound healing and reparative wound healing are that in regenerative wound healing, the current literature demonstrates a distinct lack of haemostasis and inflammatory phases-with a focus instead on proliferation and early remodelling (Singer, 2022).

Novosorb^®^ BTM is marketed as encouraging wound healing with rapid neodermis formation, with minimal scarring and contracture-analogous to regenerative wound healing. However, the current *in vivo* experimental data demonstrates that true regenerative wound healing may not be occurring at the biochemical level in wounds treated with BTM. In fact, as compared with Integra^®^, a dermal matrix containing biological collagen, BTM treated wounds experience higher levels of proinflammatory cytokine expression and greater vascularisation (Banakh et al., 2020).

While this may appear antithetical to effective wound healing, with concerns of scaring and fibrosis seen in wounds with a greater degree of inflammation, Novosorb^®^ BTM also attenuates biochemical signals that otherwise encourage scarring and contraction through its porous structure (D'Urso and Kurniawan, 2020). A major factor influencing the level of contraction seen in a wound is the rate of conversion of fibroblasts to myofibroblasts, which is primarily dependent on the mechanical forces experienced on the fibroblast at the time of wound healing (D'Urso and Kurniawan, 2020). Due to the ability for BTM to compartmentalise wound healing into numerous open cells connected via pores, myofibroblast transdifferentiation is limited. Furthermore, any existing myofibroblasts are impaired in their ability to establish contiguous collagen architecture, reducing the scar burden further (D'Urso and Kurniawan, 2020).

Therefore, while wound healing with BTM is not true biochemical regeneration, scarring and contracture is limited due to its open cell structure. Furthermore, the ability of BTM to stimulate rapid inflammation may indeed encourage a prompt formation of a well vascularised neodermis that is ideal for skin grafting. Finally, the completely synthetic nature of BTM confers it an advantage in challenging wound settings such as in necrotising soft tissue infection or chronic ulceration (SA Health, 2019).

## The plethora of BTM use cases- a review of the literature

An extensive search of PubMed and Embase with a search strategy comprised of appropriate key words and MeSH terms revealed 54 papers presenting the capacity for BTM to reconstruct extensive burns, deep soft tissue infection, non graftable wound beds, chronic wounds and other challenging wounds. The qualitative learnings from each of the papers has been presented below. Tables 1, 2. Additionally, a complete summary of all 54 papers is presented as a table within the supplementary figures.

**TABLE 1:** Papers sorted by aetiology of challenging wound.

Aetiology	References
Burns	(Abla et al., 2022; Betar et al., 2023; Concannon et al., 2023; Greenwood et al., 2020; Greenwood et al., 2018; Greenwood et al., 2016; Heard et al., 2023; Kelly et al., 2021; Larson et al., 2020; Schmitt et al., 2021; Tan et al., 2022; Tapking et al., 2024a; Telianidis et al., 2020; Gładysz et al., 2022; Tapking et al., 2024b), (Wu et al., 2022a), (Wu et al., 2022b; Lo et al., 2022; Tapking et al., 2024b; Schlottmann et al., 2022; Storey et al., 2023), (Concannon et al., 2021), (Solanki et al., 2020)
Cancer	(Shah et al., 2022; Buick et al., 2024; Sun and Tan, 2021), (Wu et al., 2022a), (Li et al., 2021), (Tapking et al., 2024b), (Storey et al., 2023; Kidd et al., 2023; Concannon et al., 2021; Solanki et al., 2020)
Infection	(Chia et al., 2024; Jennings et al., 2021; AlNafisee et al., 2022; Austin et al., 2024; Barker et al., 2022; Sreedharan et al., 2019; Wagstaff et al., 2019), (Li et al., 2021; Wu et al., 2022b; Lo et al., 2022; Tapking et al., 2024b; Schlottmann et al., 2022; Storey et al., 2023; Kidd et al., 2023; Concannon et al., 2021; Solanki et al., 2020)
Trauma	(Carrington-Windo et al., 2021; Crowley et al., 2020; Damkat-Thomas et al., 2019; Jou and Chepla, 2024; Knightly and de Blacam, 2023; Najem et al., 2024; Patel et al., 2022; Saha, 2021; Saha, 2022; Wu-Fienberg et al., 2021; Wu et al., 2022a), (Wu et al., 2022b), (Tapking et al., 2024b; Schlottmann et al., 2022; Storey et al., 2023; Kidd et al., 2023; Concannon et al., 2021; Solanki et al., 2020)
Vasculopathy	(Kuang et al., 2022; Guerriero et al., 2023; Meagher et al., 2024; Semple et al., 2022), (Li et al., 2021), (Schlottmann et al., 2022)
Miscellaneous	(Frost et al., 2022; Malkoc and Wong, 2021; Cheng et al., 2021)

**TABLE 2:** Challenging aspects of the wounds in each paper.

Challenging characteristic	References
Extensive Burn	(Abla et al., 2022; Betar et al., 2023; Concannon et al., 2023; Greenwood et al., 2020; Greenwood et al., 2018; Greenwood et al., 2016; Heard et al., 2023; Kelly et al., 2021; Larson et al., 2020; Schmitt et al., 2021; Tapking et al., 2024b), (Tapking et al., 2024a), (Telianidis et al., 2020), (Lo et al., 2022)
Infection	(Gładysz et al., 2022), (Kuang et al., 2022), (Semple et al., 2022; Chia et al., 2024; Jennings et al., 2021; AlNafisee et al., 2022; Austin et al., 2024; Barker et al., 2022; Sreedharan et al., 2019; Wagstaff et al., 2019), (Malkoc and Wong, 2021; Cheng et al., 2021; Wagstaff et al., 2015; Carrington-Windo et al., 2021), (Patel et al., 2022), (Wu et al., 2022a; Li et al., 2021; Wu et al., 2022b), (Tapking et al., 2024b; Schlottmann et al., 2022; Storey et al., 2023), (Concannon et al., 2021), (Solanki et al., 2020)
Exposed Structures	(Greenwood et al., 2016), (Gładysz et al., 2022), (Kuang et al., 2022; Guerriero et al., 2023; Meagher et al., 2024; Semple et al., 2022), (Jennings et al., 2021), (Austin et al., 2024; Barker et al., 2022; Sreedharan et al., 2019; Wagstaff et al., 2019; Frost et al., 2022), (Wagstaff et al., 2015; Carrington-Windo et al., 2021; Crowley et al., 2020; Damkat-Thomas et al., 2019; Jou and Chepla, 2024; Knightly and de Blacam, 2023; Najem et al., 2024; Patel et al., 2022; Saha, 2021; Saha, 2022; Wu-Fienberg et al., 2021), (Li et al., 2021), (Lo et al., 2022; Tapking et al., 2024b; Schlottmann et al., 2022; Storey et al., 2023; Kidd et al., 2023; Concannon et al., 2021; Solanki et al., 2020)
Challenging Anatomical Area	(Chia et al., 2024), (Barker et al., 2022), (Carrington-Windo et al., 2021), (Patel et al., 2022)

### Burns

Large total body surface area (TBSA) burns present an especially challenging wound healing problem. Most pertinent is the large degree of dermal loss, necessitating extensive skin grafting. However, this is intrinsically complicated by the lack of unaffected donor site in patients with large scale burns. In response, a total of 22 papers demonstrated the ability for BTM to effectively manage large surface area burns. These reports revealed that BTM may offer four distinct advantages to traditional skin grafting: these are: immediate and permanent cover, resistance to infection, facilitation of cultured epithelial autografts (CEAs), avoidance and reconstruction of contracture.

1. **Immediate and Permanent Cover:** The main goal of early burns management is control of burns shock-mediated by evaporative water loss. BTM serves a dual purpose as both a permanent dressing and a template through which neodermis growth is expedited. In cases of massive burns as seen in the Kelly et al., 2021 report of an 8-year-old patient with 86% TBSA burns or the Greenwood et al. description of a 34-year-old patient with 95% TBSA burns, both authors credit the ability for BTM to be rapidly and permanently applied to debrided burn wounds (Greenwood et al., 2020; Kelly et al., 2021). This ensured that patients were able to contain water losses, without the pain and infection risk of multiple dressing changes. Beyond this, BTM also was noted to be relatively resistant to sheering forces, enabling patients to engage in physiotherapy even prior to definitive skin graft coverage. This further enhanced their recovery. Both patients survived their near total body burns and on follow up presented with stable wound reconstruction without major contraction.
2. **Resistance to Infection:** Due to the crucial immunological role of the skin, a large-scale burn renders patients in a state of relative immunosuppression, leaving them susceptible to infection. Infection is deleterious to the chances of skin graft take and can pose a systemic threat to the patient. Due to the entirely synthetic nature of BTM, it less readily fosters wound colonisation and infection mediated skin graft loss as compared to biologically based dermal substitutes. Other dermal substitutes containing biological components may serve as nutrition for micro-organisms to thrive on. Therefore, infection of either a traditional skin graft or biological dermal matrix may necessitate complete removal of the material and consideration of regrafting. Thus far, this has not been the experience with BTM. Papers by Wagstaff el al, Abla et al. and Schlottman et al. describe colonisation of the BTM covered wound bed with bacteria such as *Pseudomonas* Aureginosa (Abla et al., 2022; Greenwood et al., 2018; Schlottmann et al., 2022). However, these were managed by simply evacuating any bacterial collection, cleaning the area with topical antimicrobials, and reapplying the existing matrix. The current bed of literature demonstrates that colonisation of BTM often does not require graft removal and reoperation (Abla et al., 2022; Greenwood et al., 2018; Schlottmann et al., 2022).
3. **Facilitation of Cultured Epithelial Autografts (CEA):** Scarcity of donor skin graft remains a fundamental issue in burns management. Parallel to the development of BTM and other dermal substitutes is the CEA. Using a small biopsy of the patient’s skin, a cell culture is expanded *in vitro* and prepared into sheet form or suspended within a spray. However, the integration of CEA requires the presence of dermis. Five papers demonstrate that BTM is able to facilitate the integration of CEA to a wound bed, thereby further reducing the necessity for epithelial coverage with a skin graft (Abla et al., 2022; Greenwood et al., 2020; Heard et al., 2023; Kelly et al., 2021; Larson et al., 2020). In this way, skin grafts may be meshed more liberally (up to 6:1), in order to cover greater total body surface area without extensive donor site demand (Heard et al., 2023).
4. **Avoidance and Reconstruction of Contracture:** Rapid wound healing from burns in the absence of a robust dermis results in contracture. This can be especially debilitating over mobile areas such as the neck, axilla, and groin, leading to permanent deformity. Even with traditional skin grafting, unequal provision of dermis to the wound bed can leave the wound prone to contracture. As explained above, BTM facilitated wound healing reduces the chances of inappropriate myofibroblast mediated contracture. This is reflected in the literature where most reports of extensive burns demonstrate a reconstruction minimally complicated by contracture on long term follow up. Beyond these, there are two reports of BTM being used in secondary burns reconstruction to good effect (Gładysz et al., 2022; Concannon et al., 2021).

### Complex trauma and non-graftable wound beds

Trauma presents an intricate wound reconstruction challenge due to its indiscriminate destruction of tissue layers and types. Many traumas combine damage to bone, tendon, and soft tissue, thereby requiring the reconstruction of multiple tissue types at once. Often this results in soft tissue reconstruction being required over exposed tendon or bone that are not amenable to skin grafting. The traditional solution to this is flap surgery, often necessitating microsurgical free flap reconstruction. These procedures pose a greater surgical and anaesthetic risk, incur increased donor site morbidity and may not be appropriate to older, frailer patients. To this end, BTM has been demonstrated in 18 reports to be able to reconstruct otherwise “un-graftable” wound beds.

Notably two reports by Damkat-Thomas et al. and Jou et al. demonstrated that exposed tendons that were primarily repaired or reconstructed with a tendon transfer could be covered by soft tissue facilitated by BTM (Damkat-Thomas et al., 2019; Jou and Chepla, 2024). All patients in both papers demonstrated cosmetically ideal soft tissue coverage with good tendon gliding and finger range of motion. Furthermore, the extensive 55 patient case series by Concannon et al. only containing patients whose wounds were deemed “un-graftable” demonstrated that 92% of these wounds could be effectively covered with BTM and skin grafting, sparing these patients a flap operation (Concannon et al., 2021).

Furthermore, soft tissue reconstruction over joints is also generally robust and does not restrict movement (Knightly and de Blacam, 2023).

### Necrotising Fasciitis and soft tissue infection

Necrotising Fasciitis (NF) is a rapidly life-threatening bacterial soft tissue infection that can lead to death if effective surgical source control is not established. The reconstructive challenge of NF is threefold. Firstly, large areas of soft tissue are required to be debrided away, leaving extensive soft tissue deficits. Secondly, despite aggressive surgical debridement and systemic antimicrobial therapy, NF wounds can still have subclinical colonisation of the insulting bacteria. Finally, the depth of infection seen in NF can lead to exposure of non graftable wound beds once effective surgical clearance of infection is achieved. As a result, reconstructive efforts must be able to encourage robust soft tissue regeneration while maintaining an environment that is resistant to infection.

It has already been established that BTM can foster soft tissue reconstruction that is resistant to infection over non-graftable wound beds. In addition to this, the current literature encapsulating 14 papers demonstrates that BTM can be used to reconstruct functionally important areas with appropriate bulk lost to NF. For example, a case described by Chia et al. demonstrated entire excision of the scrotum and penile soft tissue (Chia et al., 2024). These were successfully reconstructed with BTM, and split skin grafting. The reconstruction produced an aesthetically acceptable result, and the patient was able to urinate and achieve erection without issue. Beyond this, Al Nafisee et al. describe a case of NF that required circumferential debridement of a patient’s thigh to the level of the deep fascia (AlNafisee et al., 2022). The reconstructed defect while noticeably thinner than the contralateral leg demonstrated good bulk and allowed the patient to weight bear and ambulate. Finally, (Wagstaff et al., 2015) demonstrate the most impressive soft tissue reconstruction seen with BTM, where up to 24% of TBSA is successfully resurfaced using the dermal matrix (Wagstaff et al., 2019).

### Vasculopathy

Chronic diabetic and vascular ulcers are difficult to reconstruct wounds for two main reasons. Firstly, at the macroscopic level diabetic and vasculopathic patients have impaired macro and microvascular circulation, compromising the ability for the biochemical and cellular factors involved in wound healing to reach the wound. Beyond this, the cellular environment of the chronic wound is fundamentally altered to impede effective wound healing (Tarnuzzer and Schultz, 1996). Fibroblasts and keratinocytes are made senescent and chronic inflammation prevents an exit from the inflammatory to the proliferative phase of wound healing (Tarnuzzer and Schultz, 1996).

In these challenging wound settings, BTM has been shown in the early literature to represent a potential reconstructive option. While no biochemical basis has yet been described for how BTM proves useful in the chronic wound setting, six papers to date have demonstrated its efficacy in healing chronic neuropathic and vasculopathic wounds. Guerriero et al. demonstrates across 22 diabetic patients with concurrent peripheral vascular disease that BTM successfully integrated and reconstructed 65% of chronic foot ulcers (Guerriero et al., 2023). Beyond this the application of BTM also reduced the potential for amputation. The ‘Wound, Ischaemia and foot Infection’ (WIfI) score can be utilised to predict the potential for amputation extremities with chronic ulcers. In WIfI 4, the predicted rate is 35%, however in the Guerriero cohort, WIfI four patients only saw an 11% amputation rate.

### Cancer and radiation

Cancer excision can leave behind soft tissue defects that can be challenging to close. Nonetheless, currently the largest benefits of BTM in cancer reconstruction are surrounding perioperative radiotherapy. Radiotherapy is deleterious to the integration of skin grafts due to DNA damage, destruction of microvasculature and resultant inflammation and fibrosis. The use of BTM in skin cancer reconstruction is relatively novel with nine total reports describing its use.

The Buick et al. report as well as the two Sun et al. reports demonstrate successful soft tissue reconstruction with BTM post excision of squamous cell carcinoma, basal cell carcinoma and melanoma (Buick et al., 2024; Sun and Tan, 2021). The latter Sun et al. report demonstrates extensive soft tissue coverage of approximately 900 cm^2^ with BTM and split skin grafting. However, most encouragingly these reports demonstrate that BTM reconstructed wounds can tolerate post operative radiation therapy without any wound breakdown or necrosis.

At the frontier of cancer reconstruction with BTM is the report by Shah et al. which demonstrates the use of two layers of BTM to reconstruct a suprafascial abdominal wall defect left by excision of Dermatofibrosarcoma protuberans (Shah et al., 2022). The authors describe applying two layers of BTM in a stepwise fashion to offer bulk to a deep excision. At 14 months follow up, wound reconstruction is stable with a pliable scar and limited contour deformity.

### Challenging anatomical areas- scalp reconstruction

To date, scalp reconstruction has been especially challenging due to it being a highly topographic, high-tension area with a lack of tissue excess (Jang and Choi, 2020). The current literature demonstrates successful scalp reconstruction in three cases. For example, Greenwood et al., in 2016 have demonstrated successful resurfacing of a 66-year-old calvaria, lost to a burn injury (Greenwood et al., 2016). In addition to the difficult anatomical area, the scalp had lost all soft tissue and the skull had also lost all periosteum. As a result, the outer table was removed and BTM was affixed to the diploe where it successfully integrated and accepted subsequent skin grafting. The Patel et al. and Saha et al. reports confirm this within paediatric contexts as well (Patel et al., 2022; Saha, 2021).

## Conclusion

Novosorb^®^ BTM represents an exciting advancement in the realm of reconstructive surgery. It has demonstrated efficacy in challenging wound care scenarios such as extensive burn, deep set infection, poorly vascularised wound beds and in chronic non healing wounds. This is owing to its ability to act as an dermal matrix that is comparatively resistant to infection, while still encouraging effective neodermis formation and integrating successfully into the wound bed. However, the current literature, while novel, is largely single arm, low sample size and requires more robust, comparative analysis to increase confidence in BTM’s early promise.
